# Clinical Utility of IFIT Proteins in Human Malignancies

**DOI:** 10.3390/biomedicines13061435

**Published:** 2025-06-11

**Authors:** Armen Parsyan, Arpitha Kochiyanil, Anne C. Bonvissuto, Vasudeva Bhat, Alison L. Allan

**Affiliations:** 1 Department of Anatomy and Cell Biology, Schulich School of Medicine & Dentistry, Western University, London, ON N6A 5C1, Canada; 2 Verspeeten Family Cancer Centre, London Health Sciences Centre and London Health Sciences Centre Research Institute, London, ON N6A 5W9, Canada; 3 Department of Oncology, Schulich School of Medicine & Dentistry, Western University, London, ON N6A 5W9, Canada; 4 Department of Surgery, Schulich School of Medicine & Dentistry, Western University and St Joseph’s Health Care London, London, ON N6A 4V2, Canada; 5 Faculty of Science, Schulich School of Medicine and Dentistry, Western University, London, ON N6G 5C1, Canada

**Keywords:** interferon-induced protein with tetratricopeptide repeats, IFIT1, IFIT2, IFIT3, IFIT5, cancer, biomarkers

## Abstract

Interferon (IFN)-induced proteins with tetratricopeptide repeats (IFITs) are key interferon-stimulated genes (ISGs), and in humans include IFIT1, IFIT2, IFIT3 and IFIT5. These proteins are primarily known for their role in the innate immune response to pathogens. However, growing evidence suggests that IFITs participate in a range of other cellular processes, including cancer development and progression. Notably, IFITs may behave in either a pro-oncogenic or tumor suppressive fashion depending on cancer types and emphasizing their potential dual function in tumorigenesis. Importantly, IFITs have shown potential to be utilized as clinical biomarkers in oncology. Their aberrant expression has been correlated with survival and other clinical outcomes, including resistance to radiotherapy, chemotherapy, targeted treatments and immunotherapy in various cancers. Additionally, they have also been reported to be a part of various clinical predictive models in cancers. This review provides an overview of the current understanding of IFIT proteins’ involvement in cancers, with an emphasis on their emerging roles as clinically relevant biomarkers.

## 1. Introduction

Interferon (IFN)-induced protein with tetratricopeptide repeat (IFIT) genes are key interferon-stimulated genes (ISGs), including IFIT1, IFIT2, IFIT3, and IFIT5 in humans, which are known for their role in the innate immune response. IFITs, which are upregulated in response to viral infections, have been recognized for their involvement in the host defense against viruses. Beyond their well-documented antiviral activity, emerging evidence suggests that IFITs also play a significant role in the biology of cancers [1,2,3].

**Figure 1:**
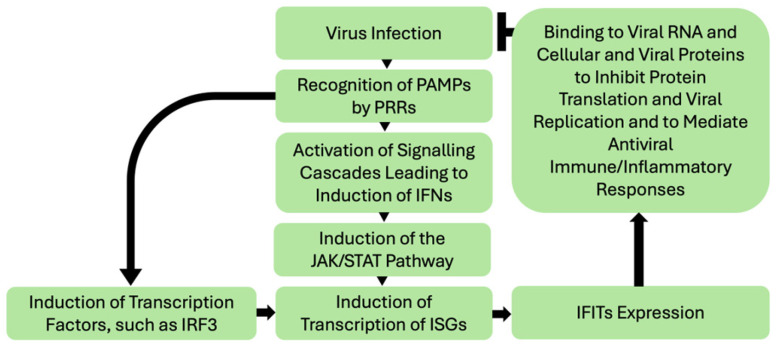
*Simplified schematic of IFITs induction by viral infections and IFITs anti-viral responses*. Viral infection leads to recognition of their PAMPs by various PRRs and subsequent activation of signaling cascades, including IFNs. IFN signaling triggers the JAK/STAT pathway leading to a nuclear translocation of the transcription factor complex ISGF3, that in turn activates transcription of ISGs, including IFITs. In the IFN-independent pathway, direct activation of factors inducing ISGs transcription can take place. IFITs then assert their antiviral function through various mechanisms, such as interacting with viral RNA and cellular and viral proteins to inhibit protein translation (namely translational initiation) and viral replication and modulating immune and inflammatory responses to viral infection. Notably, the baseline expression of IFITs in tissues is generally low and is induced in response to their activation. In addition, IFITs interactions with each other play an important role in their function [1,2,13]. PAMPs—pathogen-associated molecular patterns; PRR-pattern recognition receptors; IFN—interferon; JAK—Janus kinase; STAT—signal transducer and activator of transcription; IRF3—IFN-regulatory factor 3; ISGs—IFN-stimulated genes; IFIT—interferon induced proteins with tetratricopeptide repeats.

The details of canonical roles of IFIT proteins in anti-viral defense is well-reviewed elsewhere [2,3,4] and outside the scope of this manuscript. The generalized scheme of activation and antiviral mechanisms of IFITs is presented in Figure 1. Mammalian cells detect foreign pathogen-associated molecular patterns (PAMPs), such as in viral nucleic acids, by specific host pattern-recognition receptors (PRRs) [2]. Their binding initiates signaling to induce anti-viral response molecules such as the pro-inflammatory cytokines, type 1 interferons (IFNs). Activation of IFN signaling in response to viral infection triggers a signal transduction cascade involving Janus kinase (JAK) and signal transducer and activator of transcription (STAT) proteins, culminating in the nuclear translocation of the transcription factor complex ISGF3 (IFN-stimulated gene factor 3), comprising IFN-regulatory factor 9 (IRF9) and phosphorylated STAT1 and STAT2 [2,3]. This leads to induction of transcription of several IFN-stimulated genes (ISGs) such as IFITs. Alternatively, induction of ISGs can take place through direct activation of transcription factors such as interferon regulatory factor 3 (IRF3) in response to signaling cascades activated by PRRs [2].

IFITs then assert their antiviral function through various mechanisms, such as suppressing translation initiation, binding and sequestering viral nucleic acids and proteins and regulating immune responses—detailed mechanisms of which are reported elsewhere [2,3,4,5,6,7]. IFIT1 and IFIT2 can inhibit the translation of viral proteins by binding eukaryotic translation initiation factor 3 (eIF3) and preventing the formation of the preinitiation complex (reviewed in [2,3,5]). IFIT1 antagonizes viruses by sequestering viral nucleic acids through recognizing viral 5′-triphosphate RNA and mediating a formation of a protein complex containing IFIT2 and IFIT3 [2,8]. IFIT3 appears to modulate the activity of other IFITs and the type-I IFN response to increase the expression of ISGs [5,9]. The functional roles of IFIT5 are less well understood. It appears to play an immunomodulatory role, regulating the type-I IFN pathway [10], and NF-κB signaling [11] and potentiating anti-viral responses through enhancing innate immune signaling [12].

**Figure 2:**
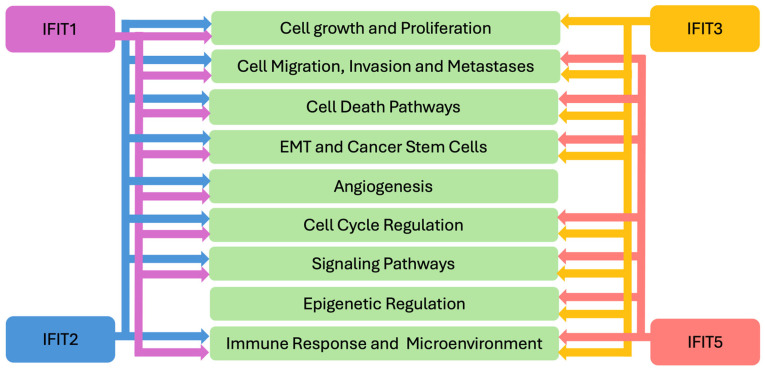
*IFITs’ Mechanisms in Cancer Pathogenesis*. EMT—epithelial-mesenchymal transition; IFIT—interferon induced proteins with tetratricopeptide repeats.

**Figure 3:**
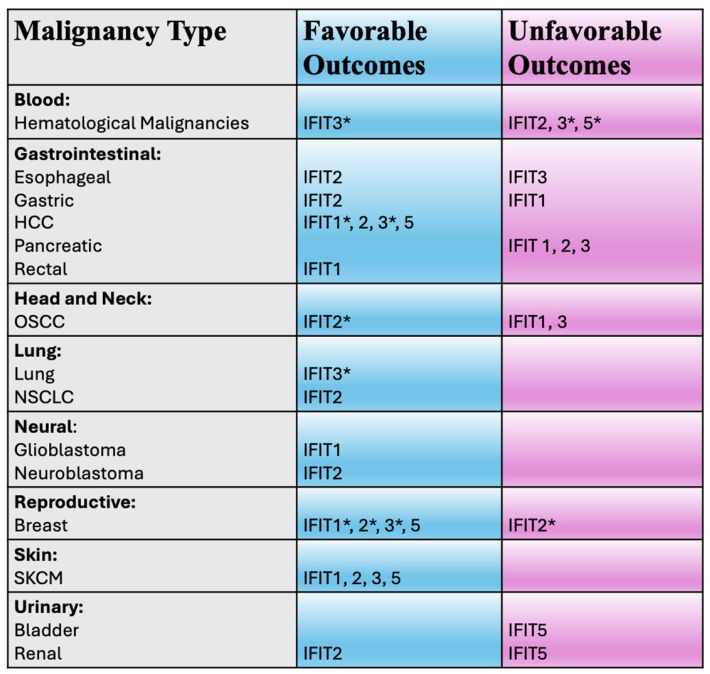
*Potential clinical utility of IFIT proteins in various malignancies*. IFIT—interferon induced proteins with tetratricopeptide repeats; HCC—hepatocellular carcinoma; OSCC—oral squamous cell carcinoma; NSCLC—non-small cell lung cancer; SKCM—skin cutaneous melanoma. * denotes that controversies exist (see text).

The role of IFITs in cancer biology is multifaceted. Emerging data indicates that IFITs participate in various biological processes, including cellular differentiation, proliferation, apoptosis, and neoplasia [1,14,15] (Figure 2). They are known to interact with key signaling pathways involved in tumor immunity, immune evasion, and the regulation of inflammatory responses. Recent studies have highlighted that IFITs’ expression can influence tumor cell progression, metastasis, and response to therapies, making these proteins important players in cancer pathogenesis and immune surveillance. As such, IFITs are emerging as potential biomarkers for cancer diagnosis, prognosis, and therapeutic response in various cancers, including hematologic malignancies, melanoma, head and neck cancers, cancer of the gastrointestinal, urinary and respiratory systems and others (Figure 3), offering promising potential for utilization in clinical decision-making in oncology. In this review, we aim to explore the current understanding of IFITs as potential clinical biomarkers.

## 2. IFIT1

The IFIT1 protein appears to exhibit either pro-oncogenic or tumor suppressor properties, depending on the cancer type. In oral squamous cell carcinoma (OSCC) [16], pancreatic [17], gastric [18] and non-small cell lung (NSCLC) cancers [19] it might promote oncogenesis and potentially associate with unfavorable clinical outcomes. Meanwhile in other cancers, such as skin cutaneous melanoma (SKCM) [20,21,22], glioblastoma [23], rectal cancer [24] and hematological malignancies [25], the opposite appears to be true. From a clinical standpoint, however, IFIT1 appears to represent a potential biomarker for prognosis, patient outcomes and treatment resistance in some cancer types (Figure 3).

### 2.1. IFIT1 and Unfavorable Clinical Outcomes

The critical pro-oncogenic role of IFIT1 in cancers is illustrated in the example of oral squamous cell carcinoma (OSCC), an anatomical subset of broader head and neck squamous cell carcinomas (HNSCC). In this cancer type, IFIT1 overexpression promotes tumor growth and metastasis through epithelial-mesenchymal transition (EMT), while its silencing leads to opposite effects, likely through enhanced p-EGFR (phosphorylated epidermal growth factor receptor) recycling [16]. Clinicopathological analysis in OSCC patients showed that IFIT1 expression positively correlated with regional lymph node invasion and nerve and venous invasion [26]. Increased IFIT1 expression also positively correlates with advanced disease stage, higher tumor grade and poor overall survival in OSCC patients [16]. In agreement with these studies, IFIT1 has also been reported to be highly expressed in HNSCC patient tissues, predicting negative clinical prognosis [27].

Furthermore, in OSCC, overexpression of IFIT1 has been reported to increase resistance to various drugs, such as cisplatin, carboplatin, 5-FU, oxaliplatin and doxorubicin; while its silencing enhances sensitivity to these chemotherapeutic agents [28,29,30]. In these malignancies, IFIT1 overexpression has also been reported to lead to resistance to targeted therapeutics, such as Hsp90 (Heat shock protein 90) inhibitors, geldanamycin and ganetespib [29]. It has been proposed that the drug resistance effects of IFIT1 are mediated through its interactions and activation of Hsp90 [28,29]. On the other hand, IFIT1 has been shown to enhance antitumorigenic effects of the EGFR tyrosine kinase inhibitor gefitinib in OSCC [16,30]. Overall, IFIT1 might have great utility as a prognostic and a predictive biomarker for chemotherapy and radiotherapy in HNSCC [14].

Similar findings have been reported in cancers of the gastrointestinal tract system, suggesting that upregulation of IFIT1 negatively influences patient outcomes and can serve as a clinical target. Upregulation of IFIT1 expression has been found in pancreatic cancer and was reported to correlate with patients’ poorer overall survival [17]. Knockdown of IFIT1 in pancreatic cancer cells decreased their proliferation, migration and invasion. The changes in these cellular processes were linked to IFIT1’s ability to regulate Wnt/β-catenin signaling and promote EMT [17]. In poorly cohesive gastric carcinomas, which are known for a poor prognosis and limited response and resistance to treatment, IFIT1 expression by tumor-associated neutrophils (TANs) promoted EMT, facilitated an exhausted phenotype of T cells and induced resistance to anti-PD1 immunotherapy [18]. Upregulation of IFIT1 by TANs facilitated gastric cancer growth, migration and invasion [18]. Consistent with these observations, the database analysis approach suggested that the high level of expression of *IFIT1* is a factor of poor prognosis in gastric cancer and correlates with poorer patient overall survival [18].

### 2.2. IFIT1 and Favorable Clinical Outcomes

In other cancer types, IFIT1 expression correlates with favorable clinical outcomes. In SKCM, elevated levels of *IFIT1* mRNA have been correlated with patients’ improved overall survival and disease-specific survival (DSS) [20]. IFIT1 also enhanced targeted therapeutics and immunotherapy in melanoma. Drug sensitivity analysis suggested that high expression levels of IFIT1 in melanoma are associated with increased sensitivity to the tyrosine kinase inhibitor, dasatinib and positively correlated with the level of immune cell infiltrates and markers [20]. IFITs were required for potentiating the responses to anti-CTLA4 and anti-PD1 immunotherapy by the Hsp90 inhibitor, ganetespib, that upregulated various interferon response genes, including IFIT1, enhancing cellular sensitivity to T-cell killing and apoptosis [21]. Melanoma patients lacking clinical responses to the anti-CTLA-4 immunotherapeutic, ipilimumab (as compared to those who had such responses) harbored a much higher rate of genomic defects in the IFN-γ pathway genes, including genetic losses of IFIT1, IFIT2 and IFIT3 [22]. This suggested that the loss of IFIT1 function might be associated with the development of resistance to immunotherapy [22]. These findings open avenues for investigations into enhancing effects of immunotherapies with combination therapies enhancing IFITs function in melanoma [21].

High expression of IFIT1 observed in 81.4% of patients with newly diagnosed glioblastoma was indicative of improved overall and progression-free survival (PFS) [23]. In patients with locally advanced non-metastatic rectal cancer, IFIT1 has been reported as a favorable predictor of response to neoadjuvant radio-chemotherapy with 5-fluorouracil (5-FU) [24].

In some cancers, such as breast cancer and hepatocellular carcinoma (HCC) the role of IFIT1 in cancer biology is controversial, with evidence somewhat skewed towards supporting its role as a favorable prognostic factor. In early-stage breast cancer patients treated with breast-conserving surgery and radiation therapy with or without chemotherapy and/or adjuvant hormone therapy, IFIT1 protein has been shown to act as a favorable prognostic marker for local control [31]. IFIT1 protein expression positively correlated with improved local relapse-free survival (LRFS), but not disease-free survival (DFS) or overall survival at 10 years [31]. Moreover, in a subset of patients with triple-negative breast cancer (TNBC), IFIT1 positivity was found to correlate with improved LRFS and DFS at 10 years, suggesting that IFIT1 may serve as a biomarker for treatment stratification [31]. However, in an earlier study, IFIT1 mRNA expression, as a part of a seven-gene-pair classifier, was found to be associated with poorer efficacy of adjuvant chemotherapy and radiation [32]. While the differences between the two studies might be explained by the differences in study designs and populations, it is important to note that Danish et al. [31] examined IFIT1 protein expression, while Weichselbaum et al. [32] used mRNA expression, the former being a better measure for the cellular functional activity of the protein. Furthermore, the Weichselbaum et al. study [32], in contrast to that of Danish et al. [31], did not independently identify *IFIT1* as a prognostic marker but rather as a part of a seven-gene classifier. While this controversy requires further studies, IFIT protein appears to act as a favorable prognostic marker in breast cancer.

Low IFIT1 mRNA expression was correlated with poor DSS in hepatocellular carcinoma (HCC) patients with chronic hepatitis [33]. Gene expression of IFIT1, as well as other IFITs, has been reported to be decreased in HCC tissues [34]. These findings suggest that IFIT1 might play tumor suppressive roles in HCC. However, Liu et al. [35] reported that mRNA levels of IFIT1 and IFIT3 were significantly upregulated in HCC tissue samples compared to para-neoplastic tissue samples, and in metastatic HCC tissue samples compared to nonmetastatic HCC tissue samples, with similar trends observed in terms of their protein levels. Furthermore, IFIT1 silencing inhibited HCC cell migration and attenuated HCC cell aggressiveness [35]. The differences in the observations made by these two groups might be explained by the differences in study designs and patient populations.

## 3. IFIT2

### 3.1. IFIT2 and Unfavorable Clinical Outcomes

Overall, more evidence supports the notion that IFIT2 acts as a tumor suppressor and its expression associates with favorable clinical outcomes (Figure 3). However, studies in acute myeloid leukemia (AML) [36] and pancreatic adenocarcinoma [37] suggest that this protein might also act as an unfavorable prognostic factor. Higher levels of *IFIT2* gene expression observed in AML served as a predictor of patients’ poor prognosis and significantly positively associated with the immune cell infiltration and immune checkpoint expression [36]. Drug sensitivity analysis suggested that high gene expression of IFIT2 in AML is associated with resistance to various drugs, but sensitivity to dasatinib [36]. Similarly, using a statistical database approach in pancreatic adenocarcinoma patients, higher expression of IFIT2 mRNA was reported to be associated with a lower rate of survival [37]. Furthermore, in this disease, IFIT2 may also play a role in the regulation of resistance to gemcitabine or paclitaxel chemotherapy [38].

### 3.2. IFIT2 and Favorable Clinical Outcomes

Pro-apoptotic and antiproliferative properties of IFIT2 have been reported in a range of cancer types, such as SKCM [21], respiratory cancers [39], gastrointestinal cancers [40,41,42,43,44], cervical cancer cells [45], leukemia cell lines [46,47] and osteosarcoma cells [48].

In addition, IFIT2 has been shown to serve as a favorable clinical indicator in several cancer types. Similarly to IFIT1, high expression of *IFIT2* in SKCM is associated with improved overall survival and DSS, sensitivity to dasatinib and immunotherapy potentiating effects of ganetespib [20,21]. Genomic defects in IFN pathway genes, including IFIT2, have been commonly observed in patients with poor responses to immunotherapy [22], suggesting that IFIT2 can be used to predict responses to immunotherapy [49] and targeted therapy.

IFIT2 expression is associated with favorable clinical outcomes in respiratory, gastrointestinal and urinary tract cancers. Decreased IFIT2 protein expression was found in both lung adenocarcinoma and lung squamous cell carcinoma, compared to the adjacent normal tissues [39], correlating with poor overall survival in NSCLC patients [39].

Higher IFIT2 expression was correlated with better overall survival in gastric cancer patients [40], where its expression was found to be significantly lower compared to adjacent normal tissues [40]. High IFIT2 expression levels were also correlated with better overall survival in esophageal cancer patients compared to those with low IFIT2 expression [41]. In lung [39], gastric [40], esophageal [41] and gastric esophageal junction cancers [42], downregulation of IFIT2 has been reported to lead to pro-oncogenic phenotypes, such as increased invasion, migration, EMT or cellular viability. IFIT2 gene expression has been reported as a favorable prognostic factor in terms of overall survival and PFS outcomes in HCC [50] patients whose tissues commonly exhibit decreased expression of IFIT2 [34]. IFIT2 upregulation through modulation of the p-STAT3-IFIT2 signaling axis by long non-coding RNA00364 has been linked to repression of HCC proliferation [43]. Although the clinical relevance of IFIT2 in other gastrointestinal tract cancers has yet to be understood, studies from these cancer types suggest that this protein behaves as a tumor suppressor. For example, in colorectal cancer (CRC) where *IFIT2* expression has been reported to be significantly lower in patients’ cancer relative to normal tissues [51], its exogenous expression led to decreased cell proliferation and increased apoptosis [52]. These tumor suppressive effects of IFIT2 in CRC have been proposed to be regulated by a blockage of its degradation by inhibition of proteasome activity and subsequent aggregation of IFIT2 in the centrosome [53] and suppression of the JAK1/STAT/IFIT2 network by the Ajuba scaffold protein [54]. In gallbladder cancer, inhibition of proliferation and metastasis by IFIT2 upregulation through transcription by PLZF (promyelocytic leukemia zinc-finger) has been reported [44]. In intrahepatic cholangiocarcinoma, IFIT2 expression has been shown to be negatively regulated through accelerated decay involving the m6A methyltransferase METTL3, that has been shown to be upregulated in this disease and to predict poor prognosis [55].

Similarly, in patients with clear cell renal cell carcinoma (ccRCC), higher IFIT2 expression correlated with improved overall survival, compared to patients with lower IFIT2 expression [56]. Weaker immunohistochemical staining of IFIT2 protein was observed in ccRCC cells compared to adjacent normal tissues [56]. A report suggests that downregulation of IFIT2 gene expression might be associated with unfavorable prognosis in neuroblastoma patients [57].

In OSCC, commonly found higher expression of IFIT2 protein in cancer tissues is associated with better patient survival, increased tumor differentiation and lower nodal stage of disease [58]. Consistent with these observations of a tumor suppressive function of IFIT2, its depletion in OSCC cells induced EMT and cancer stem cell-like phenotypes, enhanced cell migration and invasion and metastatic activity via mechanisms that potentially involve mediation of atypical PKC (Protein kinase C) signaling and TNF-alpha upregulation [58,59,60,61]. Moreover, IFIT2 depletion in metastatic OSCC cells induced muscle atrophy and cancer cachexia in mouse xenograft models by promoting IL6 (interleukin 6) production [62]. IFIT2 also appears to play an important role in modulating drug responses in OSCC. IFIT2 knockdown in OSCC cells resulted in higher resistance to 5-FU than control cells, via enhancement of thymidylate synthase expression, which mediates 5-FU resistance [63]. In another type of head and neck cancer (HNC), nasopharyngeal carcinoma, microarray data analysis indicated that the expression of *IFIT2* is downregulated in chemoradiotherapy-resistant cancer cells compared to that of sensitive cells [64]. These studies suggest that IFIT2 expression might be a favorable clinical parameter. However, a study of a small subset of patients with OSCC suggested that upregulation of *IFIT2* may promote radiation resistance and suppress the intratumoral immune response [65]. Differences in study designs and populations might account for the observed discordance in the results between these studies and warrant further investigations.

More controversies exist in terms of the role of IFIT2 in breast cancer. In TNBC, database bioinformatics analysis revealed that lower IFIT2 mRNA expression was associated with worse prognosis in terms of relapse-free survival (RFS) compared to patients with higher expression of *IFIT2* [66]. IFIT2 gene expression appears to be decreased in metastatic breast cancer compared to primary breast tumor or normal breast tissues [66]. In treatment-resistant MDA-MB-231 TNBC cells (which show enhanced migration, invasion, and stem cell-like characteristics compared to the parental line) transcriptome analysis indicated that IFIT2 gene expression was downregulated [66], suggesting its role in the modulation of treatment responses in TNBC. Reversal of IFIT2 expression by baicalein (a flavonoid with anti-inflammatory and anticancer properties) re-sensitized treatment-resistant MDA-MB-231 cells, induced apoptosis and suppressed stem cell-like characteristics [66]. These findings suggest favorable functions of IFIT2 in treatment response in breast cancer. In contrast, Gao et al. [67] proposed that overexpression of IGF2BP2 (insulin-like growth factor 2 mRNA binding protein 2) in MDA-MB-231 cells leads to increased cell proliferation and invasion by inducing expression of IFIT2 mRNA, alongside other genes. Using database analysis, the expression of IFIT2 was positively correlated with immune infiltration of the tumor and high *IFIT2* expression was associated with a decreased 5-year survival rate of breast cancer patients [67]. However, in the latter study the subtype of breast cancer was not specified. Whether these controversies between studies of Koh et al. [66] and Gao et al. [67] are related to differences in methodology and/or patient populations requires further investigation. However, the known high heterogeneity of breast cancer and molecular differences between its various subtypes might, at least in part, account for these disparate findings.

## 4. IFIT3

IFIT3 appears to act as a pro-oncogene in some cancers, such as HNSCC/OSCC [16,68]; pancreatic cancer [69] and CRC (by promoting cell viability and migration) [70]. While in other cancer types, such as SKCM [21] and prostate cancer [71], it might behave as a tumor suppressor. In other malignancies, such as hematological malignancies, HCC and lung cancer, it remains controversial whether IFIT3 acts as a pro-oncogene or tumor suppressor. Various studies suggest that IFIT3 might serve as a clinical biomarker in OSCC [16,26,72], lung cancer [73], gastrointestinal tract [50,74,75] and hematological malignancies [36,76], melanoma [20], breast [77], bladder [78] and thyroid [79] and other cancers. However, depending on the cancer type, IFIT3 expression might be associated with unfavorable or favorable clinical outcomes (Figure 3).

### 4.1. IFIT3 and Unfavorable Clinical Outcomes

In HNSCC in general [68] and OSCC in particular [16,26,72], increased IFIT3 expression, similar to IFIT1, positively correlates with unfavorable clinical and pathological characteristics, such as poor overall survival, advanced disease stage and higher grade histopathological findings. Significant upregulation of IFIT3 is observed in OSCC [72,80] and HNSCC [68] tissue samples. In this cancer type, increased IFIT3 expression promoted tumor growth, invasion, metastasis, EMT and a pro-inflammatory tumor microenvironment [16,68], as well as resistance to chemotherapy and targeted therapy [28,29,30]. Moreover, in HNSCC, IFIT3 regulates EMT and cancer stem cells by targeting PD-L1 through the PI3K/AKT pathway [68] and may participate in responses of HNSCC cells to EGFR/ERBB inhibition [81].

*IFIT3* has been proposed to be a likely biomarker of tumorigenesis for esophageal squamous cell carcinoma (ESCC) [82]. In ESCC, low versus high expression of *IFIT3* in cancer tissues was associated with prolonged DFS and overall survival [74]. These effects might be explained, at least in part, by the observation that, in this cancer, IFIT1/IFIT3+ T cells mediate immunosuppression in metastatic lymph nodes, thus allowing the propagation of the metastatic disease leading to poorer clinical outcomes [83].

In pancreatic ductal adenocarcinoma (PDAC), Zhao et al. [75], using tissue microarrays, observed shorter overall survival for patients with IFIT3 high-expressing tumors receiving adjuvant chemotherapy. To account for potential heterogeneity and to validate these results, subsequent studies from this group were then conducted using whole slides of a smaller number of patients to cover larger areas of the tumor as opposed to tissue microarrays [84]. Indeed, heterogeneous IFIT3 expression was observed in 16.4% of tumor specimens and no survival benefit for patients with tumors that expressed low IFIT3 levels was reported, with heterogeneity considered one of the potential explanations of divergent results. However, the researchers found that pancreatic cancer patients who received neoadjuvant chemotherapy had a significantly longer DFS when IFIT3 expression in their tumors was low [84]. These results prompted authors to suggest that IFIT3 might mediate chemotherapy resistance in PDAC and have a utility in predicting the success of neoadjuvant chemotherapy. IFIT3 expression might also be associated with the location of the first relapse, as patients with the first recurrence in the lungs survived longer and showed low IFIT3 expression in cancer tissues [84]. Gene array data analysis in human PDAC patient samples showed a trend of increased IFIT3 expression by ~1.5 times in samples of patients with poor outcomes, as compared to patients with good outcomes [69,85]. In line with these findings, IFIT3 was reported to be upregulated in the aggressive, versus less aggressive, pancreatic cancer cell lines [69]. Transgenic expression of IFIT3 led to increased proliferation rate, larger orthotopic tumors and higher prevalence of metastases and rescued starvation-induced apoptosis. Furthermore, in this cancer, IFIT3 expression was positively associated with increased resistance to chemotherapeutics, such as gemcitabine, 5-FU and irinotecan, through various pathways [69,75,86,87].

The role of IFIT3 in hematologic malignancies appears to be complex, where it might act as a pro-oncogene or tumor suppressor depending on the type of disease. Bioinformatics approaches have identified that higher levels of IFIT3 gene expression found in AML patient samples, predicted poor patient prognosis, associated with immunosuppressive phenotypes and resistance to various chemotherapeutics [36]. In another hematological malignancy, diffuse large B-cell lymphoma (DLBCL), decreased gene expression levels of IFIT3 were linked to enhanced immune therapy sensitivity [76]. These studies highlight potential pro-oncogenic functions of IFIT3 in hematologic malignancies.

### 4.2. IFIT3 and Favorable Clinical Outcomes

However, other studies suggest that IFIT3 can act as a tumor suppressor in hematological malignancies and is associated with favorable clinical outcomes. *IFIT3* expression levels were reported to be reduced in the peripheral blood of patients with various types of leukemia compared to healthy controls, suggesting a tumor suppressor role of this protein in hematological malignancies [88]. In patients with acute promyelocytic leukemia (APL or AML-M3) treated with all-trans retinoic acid (ATRA) that induces IFIT3 expression, high *IFIT3* expression levels in peripheral blood were found to be associated with remission, while a relapse was associated with *IFIT3* expression levels returning to lower levels, as observed before treatment [88]. Based on in silico analysis of primary central nervous system lymphoma (PCNSL) patients’ data, high IFIT3 gene expression was reported to be associated with better patient prognosis [89]. Potential tumor suppressive properties of IFIT3 in hematological malignancies are also highlighted by other studies. For example, in multiple myeloma, IFIT3 activation has been linked to repression of MYC proto-oncogene [90]. In another study in myeloma and leukemia, upregulation of IFIT3 in response to the antiparasitic drug clioquinol, was found to be essential in this drug induced pyroptosis in these malignancies [25]. Differences in results regarding the role of IFIT3 in hematological malignancies might be explained by differences in study designs, measured outputs and molecular variabilities between various types of leukemias and require further nuanced understanding.

In SKCM, low expression levels of *IFIT3,* similar to IFIT1 and IFIT2, have been found to be correlated with patients’ poor overall survival and DSS and decreased sensitivity of melanoma cells to dasatinib [20] and T-cell killing and apoptosis [21]. Genetic defects in IFIT3 have also been suggested to confer resistance to immunotherapy in melanoma [22].

IFIT3, whose expression in HCC tissue samples compared to normal ones is decreased [34,50], similar to IFIT2, has been reported to be a good prognostic factor for HCC with improved overall survival and PFS outcomes [50]. Higher expression of IFIT3, but not other IFITs, in HCC tissues predicted better response to IFN-α therapy, potentially through STAT1/2 augmentation of IFN-α effector signaling [34]. However, results from another study suggest that IFIT3 might behave in a pro-oncogenic manner in HCC [35]. In that study, IFIT3 silencing was shown to inhibit HCC cell migration and attenuate aggressiveness via complex regulation involving cancer associated fibroblasts-mediated secretion of CXCL11 and circular and micro RNAs [35]. The mRNA levels of *IFIT3* were significantly upregulated in HCC tissue samples compared with the para-neoplastic tissue samples, and in metastatic HCC tissue samples compared with the nonmetastatic HCC tissue samples, with similar trends being observed in terms of their protein levels [35]. However, this study did not investigate clinical correlations related to this observation.

IFIT3 is found to be frequently downregulated in lung cancer tissues and cell lines and is correlated with poor prognosis in patients [73]. In lung adenocarcinoma, patients’ tumor high infiltration level of IFIT3-positive neutrophils was positively related to the response to immune-targeted therapy and activation of CD8+ T cells [91]. Overexpression of IFIT3 in lung cancer leads to reduced cell proliferation, growth, migration and EMT [73,92]. Mechanisms of action of IFIT3 in lung cancer appear to be multifaceted. IFIT3 has been shown to act as a tumor suppressor by activating p53 signaling pathway in this disease [73] and by suppression of STAT3 and NF-κB [92]. Activation of the STAT1/IRF9/IFIT3 axis via FAM210B (family with sequence similarity 210 member B) has been reported to lead to the inhibition of proliferation and migration of lung adenocarcinoma cells [93]. A mechanism of regulation of IFIT3 by its direct interaction with circular RNA Circ_BBS9 has been identified to lead to tumor suppressive phenotypes modulating ferroptosis and the tumor immune microenvironment in lung cancer [94]. In contrast, in NSCLC, a report suggests that IFIT3 might, however, act to promote cancer progression via its upregulation by COL8A1 and subsequent mediation of EGFR activation, a major factor in pathogenesis of this disease [19].

In breast cancer, IFIT3 might play an important role in modulation of the immune responses to cancer cells and treatment resistance. *IFIT3* expression was reported to be higher in the peripheral double-negative T cells (a distinct subset of T lymphocytes implicated in immune responses) of breast cancer patients compared to healthy controls [77]. Furthermore, higher expression of *IFIT3* was correlated with better overall survival and immune infiltration in breast cancer patients [77]. Thus, IFIT3 may serve as a favorable prognostic indicator in breast cancer. In indirect support of tumor suppressive roles of IFIT3 in breast cancer, it has been shown that non-malignant breast cells express relatively higher IFIT3 mRNA in culture conditions, compared to MCF7 and MDA-MB-231 breast cancer cell lines [95]. A recent study has identified a specific module with the highest correlation for each breast cancer subtype, where IFIT3 was a hub gene with the highest correlation for a Basal A subtype (that often includes the TNBC subtype) suggesting its potential importance in this subtype [96]. Doxorubicin-resistant cisplatin-sensitive BrCCh1 primary breast cancer cells exhibit low baseline IFIT3 mRNA with a prominent response to stimulation with a small nucleolar RNA analogue or IFNα, suggesting that IFIT3 can function as a predictive marker for immunostimulant treatments in breast cancer [95]. On the other hand, a pro-oncogenic role of IFIT3 in mouse breast cancer models has been reported. IFIT3-positive neutrophils, that appear to play a role in the formation and development of lung metastases in breast cancer, were, in mouse models, reported to be significantly abundant in the metastatic lung microenvironment as compared to primary tumor [97]. This increased abundance was partly associated with poor prognosis, suggesting that IFIT3 might act as a pro-metastatic factor in this disease [97]. Thus, the role of IFIT3 in breast cancer might be multifaceted and requires further granular understanding.

## 5. IFIT5

IFIT5, similar to other IFITs, and depending on cancer types, elicits either pro-oncogenic or tumor suppressive functions depending on cancer types and can serve as either an unfavorable or favorable clinical marker (Figure 3).

### 5.1. IFIT5 and Unfavorable Clinical Outcomes

In bladder cancer, expression of IFIT5 has been reported to be negatively correlated with favorable pathological characteristics of the tumor and patient survival [98]. In this cancer, IFIT5 protein has been shown to induce EMT and promote cell migration/invasion via micro-RNA regulatory mechanisms and was proposed to act as an oncogene [98]. In patients with renal cell carcinoma, *IFIT5* expression is significantly higher in primary tumor tissue compared to benign tissues, and elevated levels of IFIT5 mRNA is a predictor of poor overall survival in patients [99]. IFNs can enhance renal cell carcinoma invasion via a new mechanism of IFIT5-mediated degradation of tumor suppressor miRNA, promoting EMT [99].

A pro-oncogenic role of IFIT5 was suggested in prostate cancer, where induction of IFIT5, through miRNA processing mechanisms, promoted EMT, cell invasiveness and lung metastases in vivo [100]. IFIT5 autoantibodies were upregulated in plasma samples of prostate cancer patients compared to healthy control and thus proposed to be a potential diagnostic modality in this disease [101].

Higher levels of *IFIT5* expression found in patients with AML have been shown to predict poor clinical outcomes and positively associate with immune cell infiltration and immune checkpoint expression and resistance to various drugs [36,102]. In contrast, tumor suppressive properties have been suggested in a recent study in AML, that showed that upregulation of IFIT5 can take place via regulation by histone demethylase PHF8, triggering a differentiation-apoptosis network and counteracting the growth of AML cells [103].

### 5.2. IFIT5 and Favorable Clinical Outcomes

In addition to its pro-oncogenic role, IFIT5 also appears to act as a tumor suppressor in other cancers. IFIT5 gene expression was found to be decreased in HCC tissues [34]. Bioinformatics based studies in HCC showed that higher IFIT5 expression correlates with better survival [104]. IFIT5 was proposed to be deregulated by doxorubicin treatment of breast cancer patients. IFIT5 may play a protective role in breast cancer patients [105]. Researchers identified survival-associated genes that were deregulated in doxorubicin-induced heart failure patients with breast cancer and showed that, in these patients, low levels of *IFIT5* expression were associated with shorter DFS [105]. In SKCM, the mRNA levels of *IFIT5* were found to be elevated, with their low expression correlating with patients’ poor overall survival and DSS [20]. Bioinformatics analysis identified that high *IFIT5* expression correlated with a greater survival advantage in SKCM and was incorporated into the risk stratification system for patient survival and prognosis [106,107]. Furthermore, pathway analysis suggested that IFIT5 was involved in the apoptosis pathways, EMT and cell cycle in melanoma. IFIT5 together with other IFIT members, may serve as a novel biomarker in this disease, including immunotherapy efficacy [20].

## 6. Discussion, Conclusions and Future Directions

Growing evidence suggests that IFIT proteins play important roles in cancer biology. However, these roles are diverse between various members of the IFIT family and cancer types. While IFIT2 shows characteristics of a tumor suppressor in most cancer types, other IFITs show either pro-oncogenic or tumor suppressive properties depending on the cancer type. This review also highlights that individual IFITs regulate and are regulated by various cellular processes and signal transduction pathways depending on cancer type. These observations, together with differences in the genetic background of cancer cells of various origins, might explain, at least in part, the heterogeneity of IFIT effects in cancers.

IFITs assert their pro- and anti-tumorigenic functions through targeting various processes and mechanisms related to the cancer biology. IFITs influence cancer cell growth [16] and proliferation [17,52,67,69] and cellular migration, invasion and metastasis [16,17,59,67,69,98] (Figure 2). These proteins also influence various cellular death processes, such as apoptosis [20,21,51,53,66,103]. IFIT1 and IFIT3 participate in pyroptosis [25] and IFIT3 in ferroptosis [94]. The critical role of IFITs in regulating EMT and cancer stem cells has been shown [9,16,17,20,59,61,66,98,99]. Angiogenesis, yet another important process in cancer biology, has also been shown to be influenced by IFIT2 [14,60]. IFITs have been implicated in the regulation of the DNA-damage response and the cell cycle [20,46]. For example, IFIT3 has been reported to have antiproliferative activity by enhancing the expression of p27 and p21 proteins, negative regulators of cell cycle in monocytic luekemia cells [108].

These cancer biology processes are regulated by IFITs through complex and multifaceted mechanisms, including modulation of signaling cascades. For example, IFIT1, 2 and 3 appear to be involved in AKT signaling [16,46]. IFIT1 has been shown to participate in Wnt/β-catenin [17], EFGR [16] and NF-kB pathways [21]. IFIT2 plays a role in atypical PKC (Protein kinase C) [59] and TNF-alpha pathways [14,60]. IFIT3 influences EFGR [16] and VEGF signaling [69], and IFIT5, the NF-kB pathway [11]. Furthermore, IFITs participate in epigenetic regulatory mechanisms in cancer. Some of these mechanisms involve regulation of circular RNAs by IFIT3 [94] and miRNAs by IFIT5 [98,99].

Given the important role of IFITs in anti-viral response and immunity, it is not surprising that they also influence immune-mediated tumorigenic processes, namely modulating the tumor immune microenvironment and immune cell function [9,20,21,103] (Figure 2). As part of a larger group of ISGs, IFIT expression is regulated by IFNs, critical immune-regulatory cytokines that are known to orchestrate anticancer immunity via directly impacting tumor cells or indirectly inducing the immune system [109,110]. While IFNs have been used in treatment of malignancies, IFN stimulation can lead to tumor suppressive or pro-oncogenic phenotypes [109,110,111]. In the context of IFITs, a prometastatic role of IFN-γ was described in prostate cancer cells, through promotion of EMT by the JAK/STAT1 pathway and induction of IFIT5 [100]. IFN-induced IFIT5 expression has also been shown to promote EMT and cell invasion in renal cancer [99]. Persisteent IFN-γ signaling increases STAT1-stimulated expression of various ISGs, including IFIT1, and in cancer cells, leads to dampening of the antitumor immune response and resistance to immune checkpoint inhibition [111,112]. On the other hand, stimulation of IFIT1 and IFIT3 expression by IFN-α increased OSCC cells sensitivity to gefitinib [16]. Thus, a better understanding of IFITs’ role in tumor response to IFN stimulation requires further investigation, that can lead to developing novel therapeutic approaches in cancer.

While IFITs are known to be induced by IFN and regulated by the JAK-STAT pathway [113], their expression and activity is also regulated and fine-tuned by various common and unique (to each IFIT) mechanisms in cancer, contributing to the observed diversity of their roles and mechanims in cancer biology. These mechanisms, include transcriptional, posttranslational, epigenetic and signaling pathway regulations. For example, In colorectal cancer cells, IFIT1, 2 and 3, and, in particular, IFIT2 expression appears to be regulated at the epigenetic level by histone deacetylase 2 (HDAC2) that decreases IFITs transcriptonal activity [114]. IFIT1 and IFIT2 have been shown to be negatively regulated by Wnt/β-catenin signaling in colorectal cancer models [51,52]. In certain cancer models, IFIT2 has been reported to be regulated by miRNA and long non-coding RNA mechanisms [42,48]. IFIT2 degradation is also regulated by proteasome activity [53]. In pancreatic cancer, expression of IFIT3 has been linked to regulation by the transcription factor SOX9 [69]. In AML, upregulation of IFIT5 can take place via regulation by histone demethylase PHF8 (Plant Homologous Domain Finger protein 8) [103]. Thus, while through common regulatory mechanisms a significantly positive correlation in the expression among four IFIT family members could be observed, such as in AML [36], in other contexts their expression levels might differ due to regulatory mechanisms unique to each protein and the cancer type. These reports suggest the presence of multifaceted and complex networks of IFIT regulation that, depending on the cancer molecular context, can lead to pro-oncogenic or tumor suppressive phenotypes related to these proteins.

Important from the translational and clinical perspective, IFITs play important roles in sensitivity and resistance to radiotherapy, chemotherapeutic agents, targeted treatments and immunotherapy. The IFIT proteins appear to have a great utility as biomarkers for diagnosis, prognosis and treatment responses. They are often found to be a part of predictive models for various cancers. Further studies are warranted to translate these promising findings into clinical practice in oncology and to investigate the roles of IFITs as potential clinical biomarkers in various other cancers. The role of IFITs in cancer pathogenesis and treatment responses also opens avenues for development of novel compounds modulating activities of these proteins and promoting novel therapeutic approaches in oncology. Overall, this review highlights the importance of further investigation of IFITs in cancers for translation to clinical practice to improve outcomes for cancer patients.

## Data Availability

Not applicable.
